# Fendiline inhibits proliferation and invasion of pancreatic cancer cells by interfering with ADAM10 activation and β-catenin signaling

**DOI:** 10.18632/oncotarget.5933

**Published:** 2015-09-30

**Authors:** Neha Woods, Jose Trevino, Domenico Coppola, Srikumar Chellappan, Shengyu Yang, Jaya Padmanabhan

**Affiliations:** ^1^ Department of Molecular Medicine, University of South Florida, Tampa, FL, USA; ^2^ Department of Surgery, Colleges of Medicine, Dentistry, and Public Health and Health Professions, University of Florida Health Science Center, Gainesville, Florida, USA; ^3^ Department of Anatomic Pathology, H. Lee Moffitt Cancer Center & Research Institute, Magnolia Drive, Tampa, FL, USA; ^4^ Department of Tumor Biology, H. Lee Moffitt Cancer Center & Research Institute, Magnolia Drive, Tampa, FL, USA; ^5^ Comprehensive Melanoma Research Center, Department of Tumor Biology, Department of Molecular Oncology, Department of Cutaneous Oncology, Experimental Therapeutics Laboratory, H. Lee Moffitt Cancer Center and Research Institute, Tampa, FL, USA

**Keywords:** pancreatic cancer, calcium signaling, ADAM10, cadherins, β-catenin

## Abstract

ADAM10 (A Disintegrin and Metalloprotease Domain 10) affects the pathophysiology of various cancers, and we had shown that inhibition of ADAM10 sensitizes pancreatic cancer cells to gemcitabine. ADAM10 is activated in response to calcium influx, and here we examined if calcium channel blockers (CCB) would impede ADAM10 activation and affect biology of pancreatic cancer cells. We find that the CCB, fendiline, significantly reduces proliferation, migration, invasion, and anchorage independent growth of pancreatic cancer cells. This was associated with ADAM10 inhibition and its localization at the actin-rich membrane protrusions. Further, fendiline-treated cells formed cadherin-catenin positive tight adherens junctions and elicited defective protein trafficking and recycling. Furthermore, the expression of β-catenin target genes, cyclinD1, c-Myc and CD44, were significantly decreased, suggesting that fendiline might prevent cell proliferation and migration by inhibiting ADAM10 function, cadherin proteolysis and stabilization of cadherin-catenin interaction at the plasma membrane. This will subsequently diminish β-catenin intracellular signaling and repress TCF/LEF target gene expression. Supporting this notion, RNAi-directed downregulation of ADAM10 in cancer cells decreased the expression of cyclinD1, c-Myc and CD44. Furthermore, analysis of human pancreatic tumor tissue microarrays and lysates showed elevated levels of ADAM10, suggesting that aberrant activation of ADAM10 plays a fundamental role in growth and metastasis of PDACs and inhibiting this pathway might be a viable strategy to combat PDACs.

## INTRODUCTION

Pancreatic cancer is the fourth leading cause of cancer-related deaths in the United States and shows less than 6% survival rate according to the Facts and Figures published by American Cancer Society [1]. The nucleoside analog gemcitabine remains the standard drug for chemotherapy but it increases patient survival only marginally [2] and use of newly developed combinatorial therapies have been overshadowed by toxicity issues thereby emphasizing the need for better treatment strategies [3-6].

It has been found that inhibition of L-type and store operated calcium entry (SOCE) associated channels abrogates proliferation and invasion of cancer cells, implying that calcium influx might contribute to cancer progression and metastasis [7-15]. Studies in pancreatic ductal adenocarcinoma (PDAC) cells have shown that expression of the SOCE-associated ORAI1 and STIM1 is associated with gemcitabine-resistance and their downregulation increases sensitivity to gemcitabine [16]. Another study has shown that the L-type calcium channel blocker (CCB) fendiline inhibits cancer cell proliferation, specifically in cells expressing mutant K-Ras, by altering K-Ras cellular distribution and downstream signaling [17]. Furthermore, the L-type CCBs, nifedipine, diltiazem and verapamil, have been shown to inhibit cancer cell proliferation by altering calcium activated potassium channels in PDAC cells [18]. Altogether, these data suggest that inclusion of CCBs would potentially prevent aberrant calcium signaling and inhibit proliferation and drug resistance in pancreatic cancer cells.

Recent studies from our lab have shown that inhibition of ADAM10 enhances sensitivity of cancer cells to gemcitabine [19]. ADAM10 is a sheddase that cleaves ectodomains of transmembrane proteins such as E-cadherins, N-cadherins, Notch, CD44 and amyloid precursor protein (APP), which play a significant role in proliferation, migration, invasion or stemness of cancer cells [20-37]. Since ADAM10 is activated in response to calcium influx [38, 39], we hypothesized that inhibition of calcium influx would abrogate ADAM10 activation and negate the oncogenic properties of PDAC cells. Furthermore, a search of the Total Cancer Genome Atlas (TCGA) portal showed that calcium channel subunits are altered in human PDACs, emphasizing the importance of aberrant calcium signaling in pancreatic cancer initiation and/or progression. Here we examined whether the established CCBs interfere with ADAM10 signaling and oncogenicity in PDAC cells. While the CCBs elicited an inhibitory effect on proliferation and invasion of cancer cells the concentrations required to see significant effect varied considerably. Among the CCBs fendiline and the SOCE inhibitor, SKF96365, showed the best results at a reasonably low concentration and since fendiline is already approved for treatment of angina in patients, we carried out additional studies using this agent. Our results show that fendiline treatment led to the formation of cadherin-catenin positive tight adherens junctions, reduced expression of β-catenin target genes and defective vesicular trafficking and recycling of ADAM10 and its substrates to the plasma membrane. Downregulation of ADAM10 using specific siRNAs showed inhibition of cyclinD1, c-Myc and CD44, a few of the β-catenin-TCF/LEF targets implying the significance of ADAM10 signaling in expression of these proliferation associated proteins. Furthermore, supporting the importance of ADAM10 in pancreatic cancer, we found that expression of ADAM10 is enhanced in primary PDAC tissues and TMAs. PDACs also showed enhanced expression of c-Myc, suggesting that ADAM10 activation and β-catenin-TCF signaling might be responsible for the increased expression of c-Myc and targeting specific calcium-dependent signaling mechanisms that are altered in PDAC might be a viable strategy for the treatment of pancreatic cancer. Since many of the L-type CCBs are clinically used for treatment of various cardiac abnormalities, an elucidation of their potential role in inhibition of cancer cell growth and invasion will enable the repurposing of these agents to combat PDAC.

## RESULTS

### Calcium channel blockers induce cytotoxicity in pancreatic cancer cells

**Figure 1:**
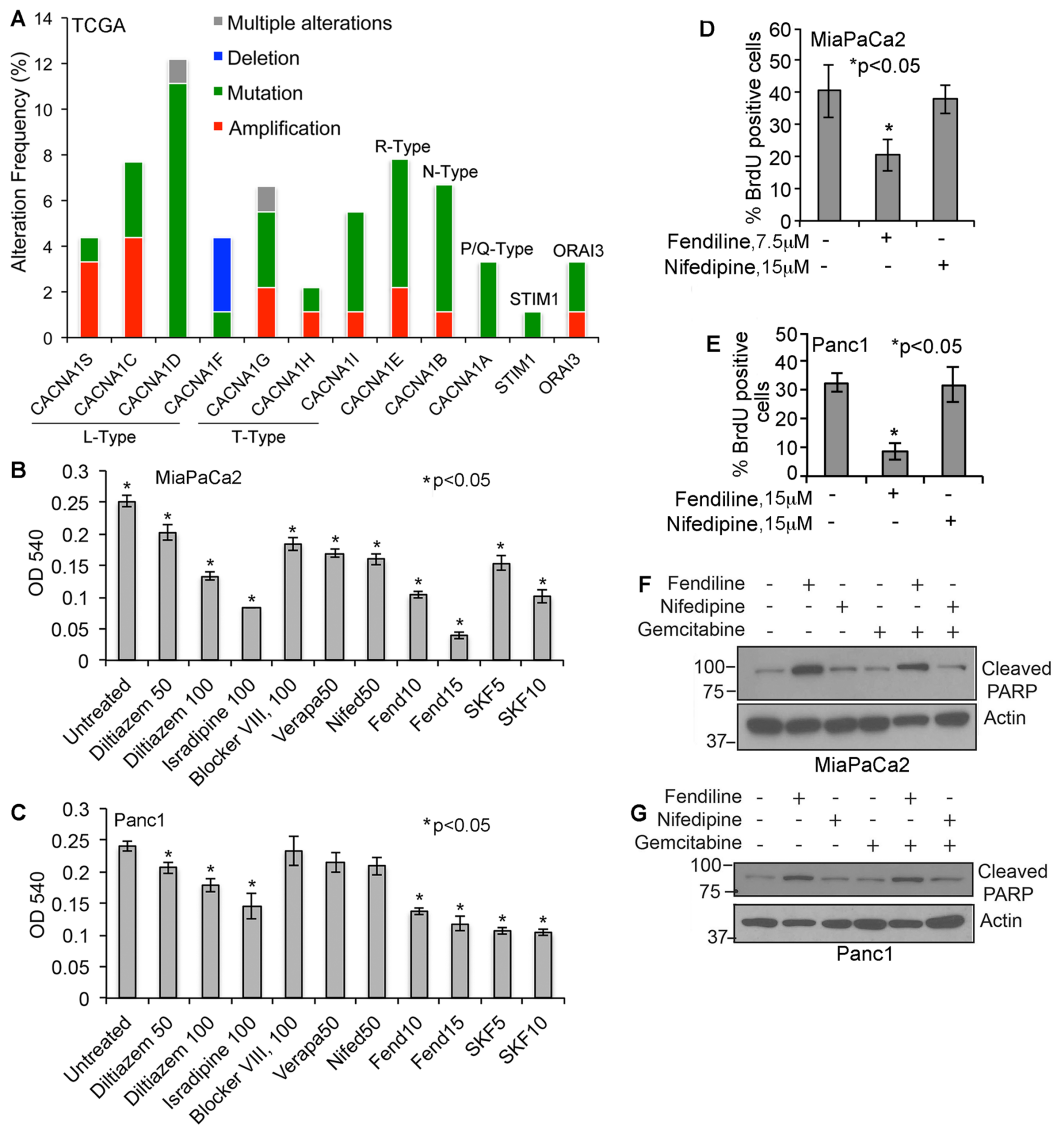
Human PDACs show alterations in voltage-gated and store operated calcium channel expression and calcium channel inhibitors induce cytotoxicity in pancreatic cancer cells **A.** Bargraph generated using the data obtained from TCGA portal shows that human PDAC samples show alterations in calcium channels. The alteration frequency in L-type, T-type, R-type, N-type and P/Q-type calcium channel subunits or ORAI3 and STIM1 are shown. **B.** & **C.** MiaPaCa2 and Panc1 pancreatic cancer cells treated with various calcium channel blockers show reduced viability. The experiments were repeated 3 times in quadruplicate and the data shows mean ± SE, *p < 0.05. **D.** and **E.** Fendiline inhibits proliferation of Panc1 and MiaPaCa2 pancreatic cancer cells: Cells were incubated with fendiline (7.5 or 15 μM) or nifedipine (15μM) for 24h and BrdU incorporation was analyzed. Experiments were repeated thrice, 100 cells were counted from 3 different areas on the slides, and the percent of cells showing BrdU positivity was calculated and plotted (mean ± SE), *p < 0.05. **F.** and **G.** Fendiline induces apoptosis in pancreatic cancer cells: Cell lysates from MiaPaCa2 and Panc1 cells treated with or without fendiline, nifedipine or gemcitabine alone or in combination were western blotted using cleaved PARP antibody. Membranes were reprobed with actin antibody for protein normalization.

Since the calcium channel blockers that we are interested in are known to elicit their effect by acting as an antagonist of voltage gated calcium influx or store-operated calcium entry (SOCE), we searched the Total Cancer Genome Atlas (TCGA) portal for alterations in these channels in PDAC [40-42]. The data showed that human PDACs show alterations in L-type (CACNA1S, CACNA1C, CACNA1D, CACNA1F) T-type (CACNA1G, CACNA1H, CACNA1I), R-type (CACNA1E), N-type (CACNA1B) and P/Q-type (CACNA1A) calcium channel subunits, as well as the SOCE-associated ORAI3 and STIM1 (Figure 1A). This suggests that aberrancies in calcium signaling might play a role in PDAC initiation and progression and that inhibition of calcium influx might prevent cancer growth. To test the effect of calcium channel inhibition, we performed studies on PDAC cell lines treated with or without CCBs. We treated MiaPaCa2 and Panc1 cells with L-type calcium channel blockers such as fendiline, nifedipine, verapapmil, diltiazem, isradipine, or the SOCE inhibitor SKF96365, which is known to inhibit STIM1 and voltage gated calcium channels. The reason for choosing Panc1 and MiaPaCa2 is that our earlier studies have shown that these two cell lines exhibit mesenchymal characteristics and are more resistant to treatment with gemcitabine compared to CD18 cells, which is epithelial in nature [19]. Studies using 1μM to 100μm of the various CCBs showed that most of them reduced cell viability only at relatively high concentrations (50-100μM) except for fendiline and SKF96365, which showed significant effects at 15μM and 10μM, respectively (Figure 1B and 1C). The exact nature of the different voltage gated calcium channels expressed by Panc1 or MiaPaCa2 cells are unknown. Studies by others have shown that Panc1 cells express the L-type calcium channel subunit CACNA1C (Cav1.2) [43] and MiaPaCa2 express T-type calcium channels [44]. Also, studies using both L-type (verapamil, diltiazem and nifedipine) and T-type (phenytoin) calcium channel antagonists have shown anti-proliferative effects on these cancer cells implying that they express voltage gated calcium channels [7, 18]. Since we observed best results with fendiline, which has been approved for treatment of angina in cardiac patients [45, 46], we performed further studies using this calcium channel antagonist.

### Fendiline enhances cytotoxicity and inhibits proliferation of cancer cells

To determine if the CCBs enhance sensitivity of cancer cells to gemcitabine, MiaPaCa2 and Panc1 cells were treated with 15μM fendiline, 15μM nifedipine, 100ng/ml gemcitabine or a combination of these drugs for 24h, and cell viability was assessed. Nifedipine at 15μM did not have any effect by itself or in combination with gemcitabine. At the same time, treatment of cells with fendiline induced significant cytotoxicity but co-treatment with gemcitabine and fendiline did not have an added cytotoxic effect, suggesting that fendiline is capable of inducing significant cytotoxicity by itself (data not shown). To assess whether fendiline or nifedipine affects cell proliferation, BrdU incorporation assays were performed. Analysis of Panc1 and MiaPaCa2 cells treated with 15μM fendiline or nifedipine for 24h showed that fendiline could significantly inhibit the proliferation of both cell types, whereas nifedipine at this concentration was ineffective. MiaPaCa2 was found to be more susceptible to fendiline than Panc1, since 7.5μM fendiline was sufficient to effectively inhibit cell proliferation as compared to 15μM of the drug used in Panc1 cells (Figure 1D and 1E). Western blotting using an antibody to cleaved PARP showed that cells treated with fendiline show increased PARP cleavage in MiaPaCa2 and Panc1 cells, indicative of apoptosis (Figure 1F and 1G), whereas nifedipine had only a minimal effect; we did not observe any increase in PARP cleavage upon co-treatment of cells with fendiline and gemcitabine, indicating that these two drugs do not show additive or synergistic effects. All together, these data suggest that fendiline exerts significant cytotoxic effects on pancreatic cancer cells and would potentially be beneficial as a single agent or in combination with other chemotherapeutic drugs in treating pancreatic cancers that do not respond to gemcitabine therapy.

These results show that although CCBs induce cytotoxicity in pancreatic cancer cells, their efficacy vary significantly. The L-type CCBs we tested belong to the dihydropyridine (eg: nifedipine and isradipine), non-dihydropyridine (phenylalkylamines, eg: fendiline and verapamil) or benzothiazepine (diltiazem) class. Fendiline is a lipophilic calcium antagonist and is shown to bind both calcium channels and calmodulin with similar affinities [45]. Although fendiline elicits similar potencies as nifedipine and verapamil under certain situations, chronic exposure to fendiline has been shown to enhance its anti-anginal effect, indicating that these drugs act differently. It is possible that this effect of fendiline is brought about by either a calmodulin-mediated mechanism or through its stabilization by incorporation in to the membrane lipid bilayer [47].

### Fendiline treatment induces G1 arrest in pancreatic cancer cells

**Figure 2:**
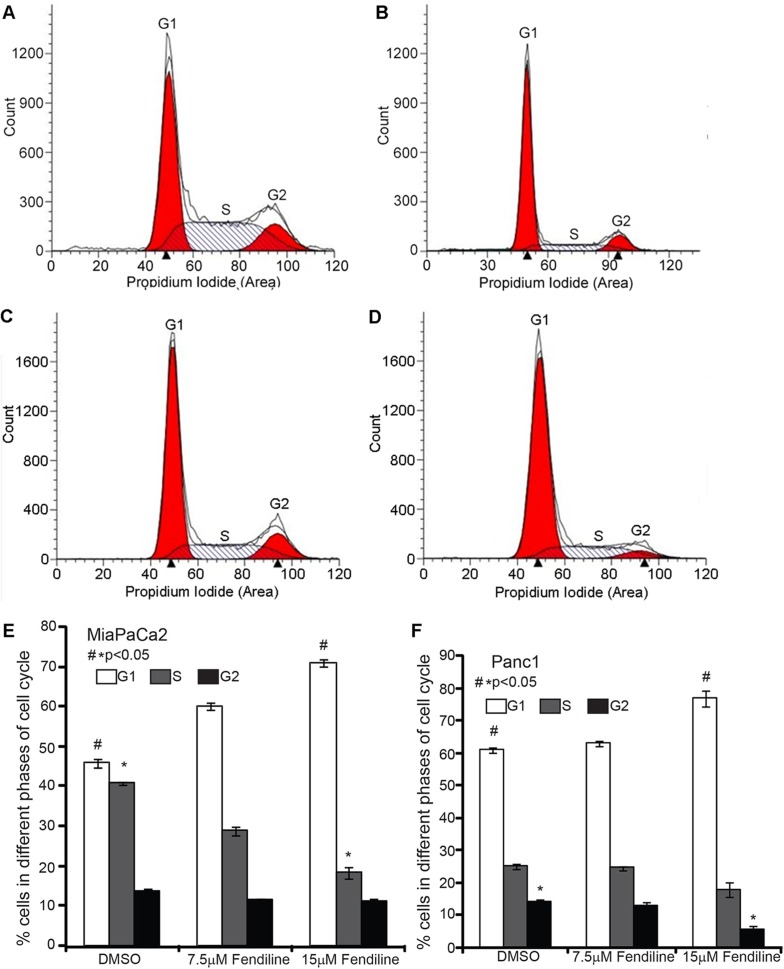
Fendiline induces G1 arrest in pancreatic cancer cells MiaPaCa2 and Panc1 cells were treated with 7.5 or 15 μM fendiline and fixed and stained using propidium iodide prior to analysis by flow cytometry. **A.**-**D.** Representative ModFit LT modeling of FACS data from MiaPaCa2 (**A.** untreated, **B.** fendiline 15μM) and Panc1 cells (**C.** untreated, **D.** fendiline 15μM). **E.** and **F.** Percent of MiaPaCa2 **E.** and Panc1 **F.** cells in G1, S and G2/M phase of cell cycle. The experiment was repeated 3 times and the average and standard deviation were used to generate the bargraph, *p < 0.05.

Since BrdU analysis showed reduced cell proliferation upon fendiline treatment, we performed propidium iodide staining followed by FACS analysis to assess changes in the cell cycle. Cells were trypsinized and cultured for 24h prior to treatment for 24h. It was found that treatment of MiaPaCa2 (Figure 2A, 2B and 2E) and Panc1 (Figure 2C, 2D and 2F) cells with fendiline for 24h resulted in significant enrichment of cells in the G1 phase. There was a corresponding reduction in the number of cells in S and G2 phases, suggesting a G1/S arrest (Figure 2E and 2F). These results, along with the data from BrdU incorporation studies suggest that fendiline inhibits PDAC cell proliferation by inducing G1 arrest.

### Calcium channel inhibition prevents anchorage independent growth, migration and invasion of cancer cells

**Figure 3:**
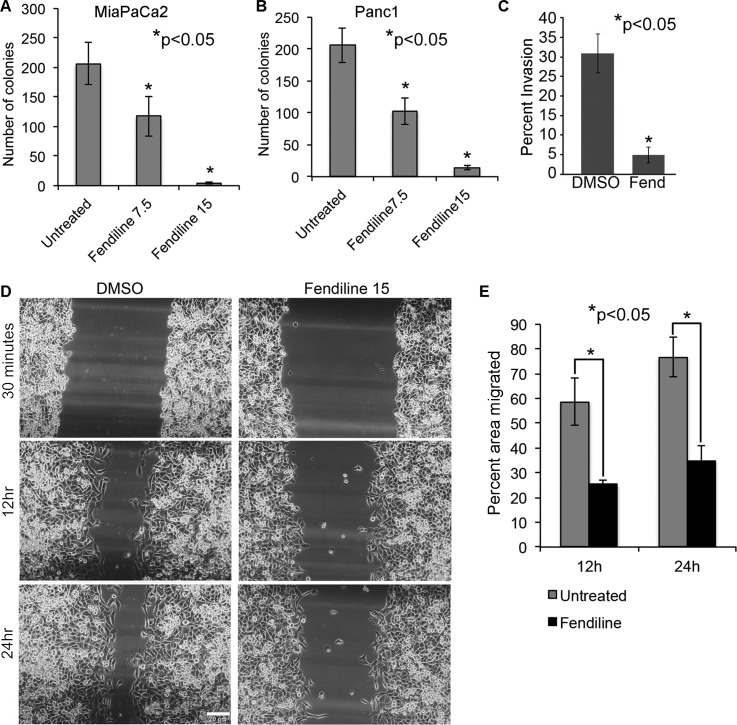
Fendiline inhibits anchorage-independent growth, migration and invasion of cancer cells 5,000 MiPaCa2 **A.** or Panc1 **B.** cells were seeded in 0.3% agar containing medium and layered on top of 0.6% base agar layer in 12 well plates. Colonies were allowed to grow in the presence or absence of 7.5 and 15 μM fendiline for 2.5 weeks and stained with MTT. Quantification was performed using ImageJ image analysis tool and the number of colonies within a defined area is counted and plotted. Each treatment was performed in triplicates and each experiment was repeated thrice (*p < 0.05). **C.** Boyden chamber analysis show reduced invasion of Panc1 cancer cells upon treatment with 15μM fendiline (Fend), *p < 0.05. **E.** Fendiline inhibits migration of cancer cells: Panc1 cells grown to confluence in a 12 well dish was growth arrested by serum starvation for 24h and scratch wounds were made. Images of treated and untreated wells were taken within 30min. after the wounds were made and at 12h and 24h periods. **E.** The cell-free area of the wound was measured and percent area covered by cells was calculated based on the initial wound area measured within 30min. Experiment was done in triplicate and repeated thrice and the bargraph shows that cell migration is significantly reduced by fendiline treatment at 12 and 24h (mean ± SD).

A colony formation assay in soft agar was conducted to determine if fendiline could affect the anchorage-independent growth of MiaPaCa2 or Panc1 cells. 5000 cells per well were seeded and treated with 15μM fendiline for 2.5 weeks and analyzed for colony formation. Fendiline treatment greatly reduced the number of colonies formed as compared to untreated cells (Figure 3A and 3B), indicating the ability of fendiline to inhibit the adherence independent growth of pancreatic cancer cells. Given these results, we examined whether fendiline affects cell migration and invasion. A Boyden Chamber Assay performed on Panc1 cells showed that treatment with fendiline drastically reduces the invasion of cells, compared to untreated controls (Figure 3C); invasion was also abolished in MiaPaCa2 cells (data not shown). To examine migration, a wound-healing assay was performed on Panc1 or MiaPaCa2 (data not shown) cells grown to confluence in 12 well dishes. Cells were treated with or without fendiline and wound area was measured within 30min. after making the wound, and after 12h and 24h. Data presented in Figure 3D and 3E show that fendiline treatment significantly reduced the migration, as measured by the percentage of the uncovered wound area in comparison to the initial area of the wound measured at 30min. These results support the anti-proliferative and anti-invasive effects of fendiline on pancreatic cancer cells.

### Fendiline promotes tight cadherin-catenin positive adherens junction formation and inhibits expression of β-catenin target genes

**Figure 4:**
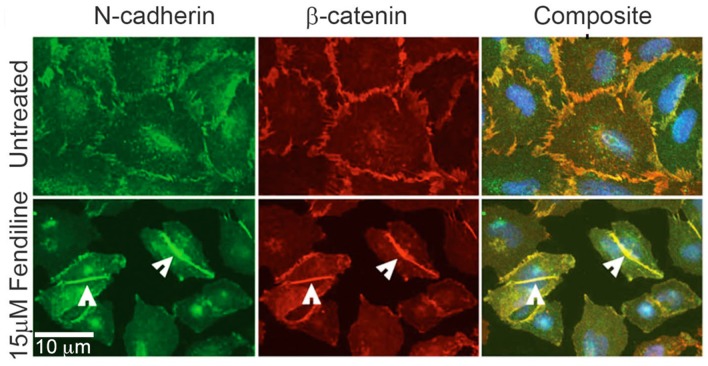
Calcium channel inhibition enhances cadherin-catenin co-localization at the adherens junctions Panc1 cells were treated with or without fendiline 15μM and immunostained using N-Cadherin and β-catenin antibodies. Alexa Fluor 488 and 594 were used as secondary antibodies and Hoechst was used for nuclear staining. Cells treated with fendiline showed enhanced colocalization of cadherin and catenin at the adherens junctions (arrow heads in bottom rows). Magnification: 63X.

Pancreatic cancer cells are known to undergo EMT (Epithelial to mesenchymal transition), which promotes their invasive and metastatic potential [48, 49]. Western blot analysis have shown that Panc1 cells express N-Cadherin [19]; fendiline treatment did not significantly increase the expression of E-cadherin in MiaPaCa2 or Panc1 cells as analyzed by real time PCR (data not shown). This indicates that the reduced migration might not be brought about by enhanced expression of E-cadherin. To assess whether fendiline affects cellular distribution of N-cadherin, we performed immunostaining analysis on Panc1 cells; for all the immunostaining analyses we chose these cells due to their flat morphology compared to MiaPaCa2 cells. Cells were treated with 15μM fendiline for 24h, fixed and analyzed using N-cadherin and β-catenin antibodies. Results showed enhanced co-localization of N-cadherin with β-catenin at the membranes and at the adherens junctions, with a concomitant reduction in the intracellular levels of β-catenin (Figure 4), indicative of stabilization of the catenin at the adherens junctions. Fendiline treated wells showed a significant reduction in cell number and the cells appeared to form tight adhesion between adjacent cells, suggesting that, in addition to inhibition of proliferation, fendiline enhances intercellular adhesions, contributing to reduced migration and invasion.

**Figure 5:**
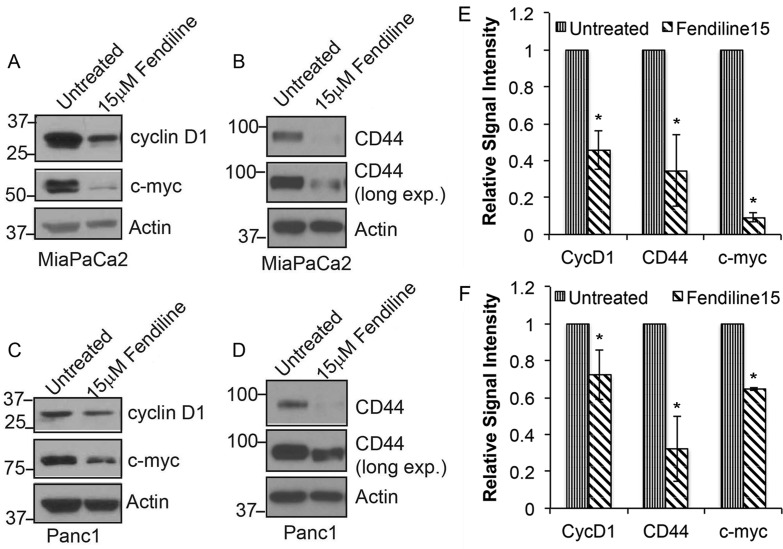
Fendiline inhibits expression of c-Myc, cyclin D1 and Cd44 MiaPaCa2 **A.** and **B.** and Panc1 **C.** and **D.** cells were treated with or without 15μM fendiline and analyzed by western blotting for cyclin D1, c-Myc and CD44 expression levels. Actin was used as loading control in each case. **E.** and **F.** Blots from 3 independent experiments using MiaPaCa2 **E.** or Panc1 **F.** cells were scanned and quantified using Image J Image analysis tool and normalized to actin levels prior to plotting, *p < 0.05.

Given the reduction in the levels of β-catenin upon fendiline treatment, additional experiments were conducted to assess the expression of β-catenin target genes. β-catenin is known to interact with T-cell factor (TCF) / Lymphoid-enhancing factor (LEF) family of transcription factors to induce gene expression [22, 50-53]. Western blotting conducted on MiaPaCa2 and Panc1 cells showed that expression of the β-catenin targets - cyclin D1, c-Myc and CD44 - was reduced upon fendiline treatment (Figure 5A-5F). These results agree with the enhanced membrane stabilization and reduced nuclear localization of β-catenin in fendiline treated cells. This also indicates that fendiline affects multiple aspects of pancreatic cancer cell biology, including signaling events mediated by β-catenin.

### Fendiline affects cellular distribution and function of ADAM10

**Figure 6:**
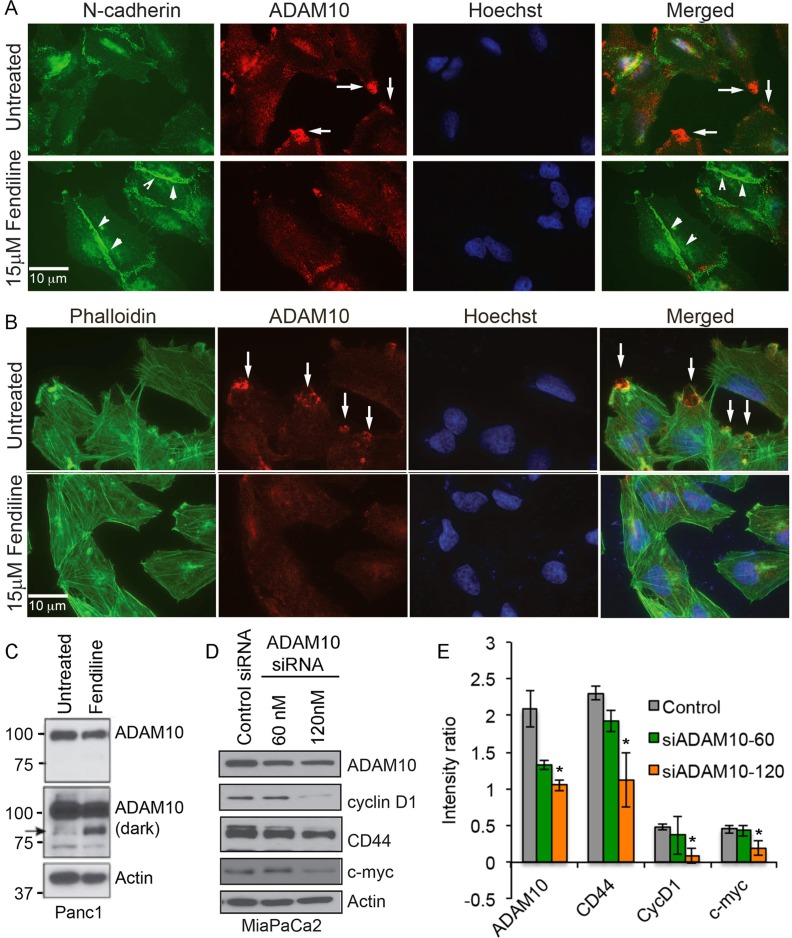
Fendiline treated Panc1 cells show reduced localization of ADAM10 at the membrane protrusions Panc1 cells were treated with or without 15μM fendiline and stained with ADAM10 and N-cadherin **A.** or ADAM10 and Alexa Fluor 488-phalloidin **B.**. Untreated cells show strong localization of ADAM10 at the membrane protrusions, as indicated by arrows in **A**. and **B**. and the arrowheads in **A.** show strong staining for N-cadherin at the cell-cell adherens junctions. Magnification: 63X. **C.** Panc1 cells treated with fendiline show a reduction in the ADAM10 levels and formation of the ∼85kDa fragment, indicated by arrow. **D.** and **E.** ADAM10 knockdown in MiaPaCa2 cells leads to downregulation of cyclin D1, CD44 and c-Myc. Blots from two independent experiments were scanned and used for quantification.

To determine if fendiline affects the levels or distribution of ADAM10, immunocytochemistry as well as western blotting was done on Panc1 cells. Since we observed an increase in the levels of N-cadherin at the adherens junctions (Figure 4B), Panc1 cells were treated with or without 15μM fendiline for 24h and co-stained with N-cadherin and ADAM10 antibodies. ADAM10 was heavily localized at the plasma membrane protrusions in untreated cells, indicative of its role in cell detachment and migration (arrows in Figure 6A, top panel). N-cadherin staining was reduced in the areas of strong ADAM10 localization, possibly due to proteolytic processing of N-cadherin by ADAM10. Fendiline treatment reduced the localization of ADAM10 at the leading edges of the cells and increased the levels of N-cadherin at the intercellular adherens junctions (arrowheads in Figure 6A bottom panel). This indicates that fendiline-mediated inhibition of ADAM10 interferes with cadherin cleavage and stabilizes its membrane association.

Since membrane protrusions are regions of dynamic actin reorganization that contribute to cell motility and migration [54], association of ADAM10 with these leading edges suggests that it might play a role in ECM dissolution, cell detachment and movement. To confirm that ADAM10 indeed associates with the ruffles at the membrane protrusions, cells were treated with or without fendiline for 24h and co-stained with Alexa Fluor 488-labeled Phalloidin and ADAM10 antibodies (Figure 6B). ADAM10 showed strong staining at the actin-rich dorsal and ventral ruffles at the membrane protrusions (arrows in Figure 6B) and this association was significantly reduced by fendiline. This suggests that fendiline treatment leads to the inhibition of ADAM10 localization at the cell membrane, compromising its function as a sheddase and abrogating ECM degradation, cell migration and invasion.

Our published studies have shown that ADAM10 inactivation is associated with generation of an ∼85kDa fragment of the protease [19]; as shown in Figure 6C, cells treated with fendiline showed generation of the ∼85kDa ADAM10 fragment, indicative of its inactivation. The results described so far suggest that fendiline inhibits ADAM10 function, increases cadherin association at the cell membrane, and enhances cadherin-catenin positive adherens junction formation, with a corresponding decrease in nuclear levels of β-catenin. Since ADAM10 is known to enhance ectodomain shedding of cadherins, these results suggest that fendiline inhibits β-catenin signaling by interfering with ADAM10 mediated cadherin cleavage and release of β-catenin. To test this possibility, and to determine whether ADAM10 affects β-catenin signaling, we downregulated ADAM10 in MiaPaCa2 by transfecting an ADAM10 siRNA; cells transfected with a non-targeting siRNA served as control. After confirming ADAM10 downregulation by western blot analysis (Figure 6D and 6E), blots were reprobed with c-Myc, cyclin D1 as well as CD44, all of which showed reduction in ADAM10 depleted cells, indicating that ADAM10 regulates catenin-dependent intracellular signaling in the cancer cells.

### Fendiline interferes with intracellular protein sorting and recycling

**Figure 7:**
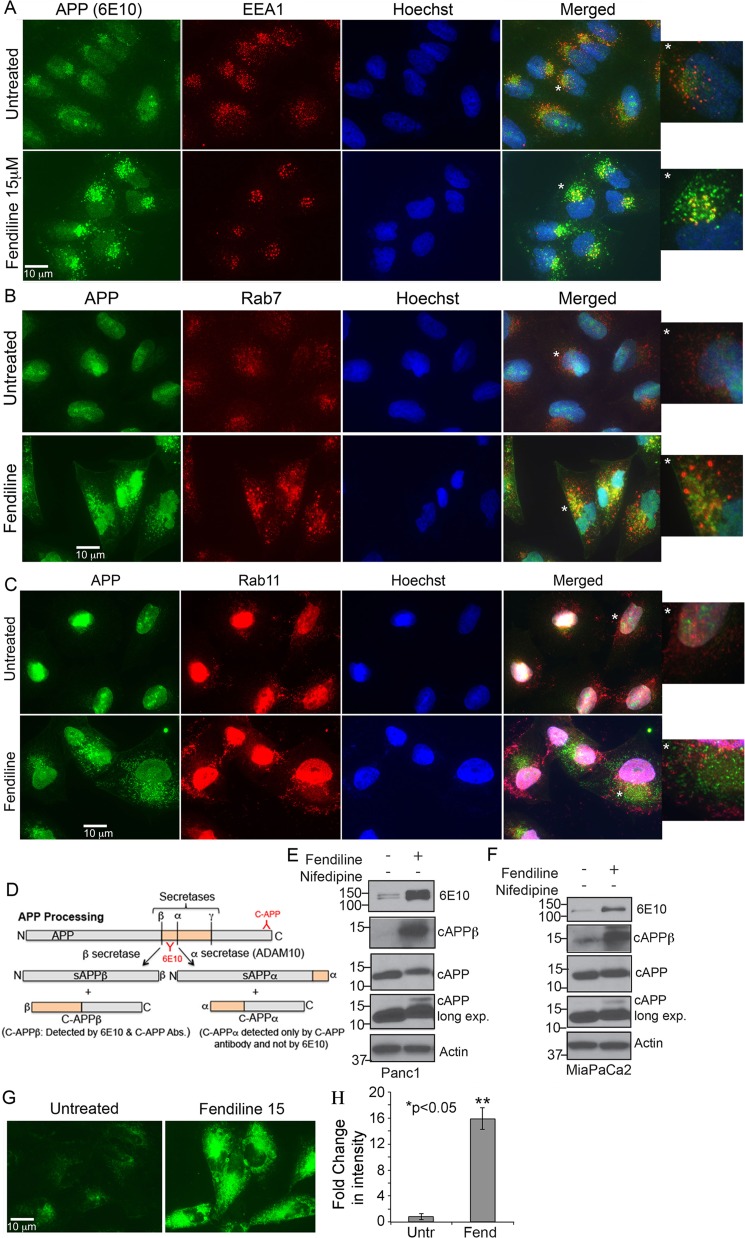
Fendiline treatment interferes with the intracellular vesicular transport Panc1 cells treated with 15μM fendiline were analyzed for changes in cellular distribution of the ADAM10 substrate APP using 6E10 antibodies and markers of endocytic pathway - EEA1 **A.**, Rab7 **B.** or Rab11 **C.** antibodies. Alexa Fluor 488 (green) and 594 (red) secondary antibodies were used for visualization of the staining and Hoechst was used as a nuclear stain. The images to the right of each panel show enlarged images of the vesicles in cells depicted by a star. Magnification: 63X. **D.** Schematic showing APP processing by secretases; ADAM10 has been established as the main α-secretase that constitutively cleaves APP. **E.** and **F.** Analysis of cell lysates from fendiline treated Panc1 and MiaPaCa2 cells show altered processing of APP upon treatment with fendiline, with enhanced β-secretase cleavage to generate the ∼12kDa C-APP fragment that can be detected by 6E10 antibodies (cAPPβ, second panel in **E.** and **F.**). Analysis using the C-APP antibodies show a slight shift (slower migration) in the C-terminal fragment in fendiline treated samples, which confirms the increased processing of APP by β-secretase rather than α-seceratse (ADAM10) (3^rd^ and 4^th^ panels in E and F). Reprobe of the blot with actin was performed to determine protein loading on the blots. **G.** and **H.** Analysis of Panc1 cells using the lipophilic FM 1-43 FX dye shows accumulation of enlarged multivesicular bodies upon treatment with fendiline, indicative of defective vesicular trafficking and recycling.

Our studies have shown that inhibition of ADAM10, which is established as the -secretase that constitutively cleaves APP, interferes with APP proteolysis [19]. APP is a transmembrane protein, which is cleaved by α, β and γ-seceretases to generate fragments with variable cellular functions [55-57]. Since fendiline seems to inhibit ADAM10, and because APP is known to play a role in PDAC cell proliferation [19], we examined if cells treated with fendiline show alterations in APP. Immunostaining analysis on Panc1 cells showed that fendiline treatment led to the accumulation of APP in large intracellular vesicles, which were positive for EEA1, an early endosomal marker (Figure 7A). There was also increased co-localization of APP with Rab7 (Figure 7B) but not with Rab11 (Figure 7C), which is a late endosomal marker that is involved in membrane fusion or exocytosis of endocytosed membrane proteins. This indicates that fendiline affects endosomal protein sorting, and inhibits vesicular trafficking and recycling of APP to the plasma membrane. At the molecular level, intracellular levels of full length APP (APP_FL_) were elevated in fendiline treated cells, indicative of reduced exocytosis (Figure 7E and 7F). 7D shows a schematic of APP proteolysis and expected fragments. It is known that endosome-associated APP is processed more effectively by β-secretase and those associated with the plasma membrane by α-secretase [58, 59]. Analysis of APP by western blot using 6E10 antibody showed an increase in the β-secretase cleaved C-terminal fragment of APP, namely cAPPβ, which is recognized by 6E10 antibodies (Figure 7E and 7F). This altered APP processing was further confirmed by western blot analysis using a C-terminal APP antibody, which showed a slight shift in migration of the C-terminal fragment (labeled cAPP), indicative of altered processing and generation of C99 (β-secretase cleaved C-terminal fragment) versus C83 (α-secretase cleaved C-terminal fragment).

To confirm the effect of fendiline on endosomal vesicle formation and sorting, Panc1 cells were stained with FM1-43FX, a membrane probe for activity-dependent vesicle cycling [60]. Fluorescence microscopy of cells incubated with FM1-43FX and treated with fendiline for 6h showed a significant increase in the number and size of FM1-43 positive intracellular vesicles (Figure 7G and 7H). This provides additional support to the contention that fendiline affects endosomal sorting and recycling, leading to the altered cellular distribution and accumulation of APP in the intracellular vesicles.

### PDAC human samples show increased expression of ADAM10

**Figure 8:**
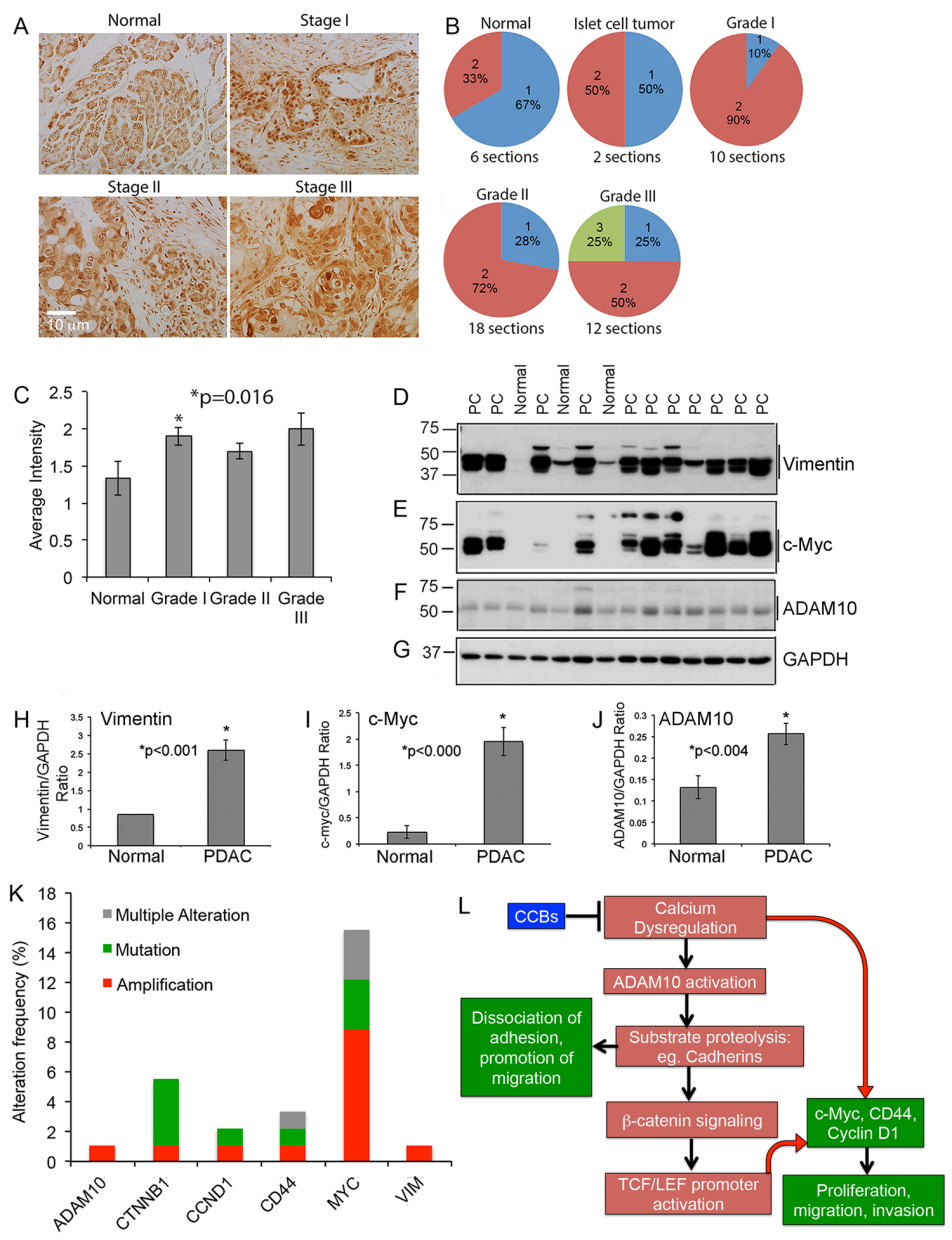
PDAC tumor tissue array show enhanced expression of ADAM10 **A.**-**C.** Tumor tissue array containing normal, islet cell tumor and Grade I, II and III PDAC tissue samples were immunostained using an ADAM10 antibody **A.** and intensity of the stained sections were measured by Dr. Coppola, Senior Pathologist at Moffitt Cancer Center. The stain was semiquantitatively scored based on the intensity of the stain as negative (0), weak (1), moderate (2) and strong (3). In all cases at least 34% of the tumor was positive, which is shown in **B.** The bargraph in **C.** shows that ADAM10 levels are increased in tumor tissues, with Grade 1 tumors showing a significant increase. **D.**-**J.** Expression of vimentin, c-Myc and ADAM10 are significantly increased in PDAC: PDAC tissue samples and samples from normal pancreas were analyzed by western blot using vimentin (**D.** and **H.**), c-Myc (**E**. and **I**.) and ADAM10 (**F.** and **J.**) antibodies and blots were reprobed with GAPDH antibody for normalization of proteins. **K.** Graph plotted using the data derived from TCGA portal show that PDAC human samples show increased alterations, especially amplification and/or mutation, in ADAM10, β-catenin (CTNNB1), cyclin D1 (CCND1), CD44, Myc (MYC) and vimentin (VIM). **L.** Proposed signaling mechanisms by which calcium dysregulation enhances ADAM10-mediated tumor progression: Based on our data with fendiline we hypothesize that calcium influx induces ADAM10 activation, leading to enhanced cadherin cleavage, release of β-catenin, its nuclear translocation and activation of TCF/LEF containing promoters. This enhances expression of genes associated with proliferation, epithelial mesenchymal transition and metastasis of cancers such as c-Myc, cyclin D1 and CD44. Additionally, β-catenin/TCF signaling has been shown to enhance ADAM10 expression thereby playing a feed-forward role in ADAM10-mediated downstream signaling and promotion of oncogenic cycle. In addition to this indirect activation of β-catenin-TCF signaling, ADAM10-mediated cleavage of substrates such as cadherins and CD44 allow detachment of cell-cell and cell-substratum adhesions, migration and invasion of cancer cells. Our data indicate that inhibitors of calcium channels prevent ADAM10-dependent signaling and expression of c-Myc, cyclin D1 and CD44, by stabilizing cadherin-catenin interaction at the cell membrane, enhancing adherens junction formation, subsequently reducing p-catenin-TCF/LEF signaling and target gene expression.

Since ADAM10 appears to affect cell proliferation and migration, we examined if ADAM10 is altered in PDACs. Towards this, we analyzed ADAM10 levels by immunohistochemistry in human PDAC tissue microarrays that contained duplicate samples representing normal pancreas, islet cell tumor, and Stage I, II and III adenocarcinomas with regional or distant metastasis. ADAM10 was elevated in the tumor samples, with grade I adenocarcinoma showing a significant increase (p=0.016); Grade II and III tumors showed an increase, which did not reach significance (Figure 8A-8C). This suggests that ADAM10 activation might play a role in tumor initiation, subsequently enhancing proteolysis of substrates that could promote tumor progression and metastasis.

Additionally, we analyzed the levels of vimentin (Figure 8D and 8H) and c-Myc (Figure 8E and 8I) in tumors from 21 PDAC patients, or normal pancreas form 5 patients by western blotting; their expression was significantly elevated in PDAC compared to normal tissues. Additionally, these samples also showed significantly higher levels of the ∼60kDa mature ADAM10 protein (Figure 8F and 8J), and comparable levels of GAPDH (Figure 8G). Since treatment with CCB and ADAM10 downregulation reduce c-Myc levels, it is possible that the increase in c-Myc observed in pancreatic cancer tissue samples is at least partly brought about by enhanced calcium signaling and/or ADAM10 activation. Furthermore, TCGA data portal (Figure 8K) shows that human PDACs exhibit amplifications and mutations in ADAM10, MYC, VIM (vimentin), CD44, CCND1 (CyclinD1) and CTNNB1 (β-catenin), implying the importance of interfering with the signaling associated with these in prevention of pancreatic cancer.

## DISCUSSION

While many CCBs are routinely used to treat cardiac diseases, their role as anti-cancer agents is far less understood. The results presented here show that the CCB fendiline can manipulate the biology of pancreatic cancer cells by affecting the cellular distribution of the proteolytic enzyme ADAM10 and its substrates. The biological function of ADAM10 as an α-secretase that cleaves APP [26, 28] as well as cadherins, notch and CD44 is well established [21-25, 27, 29]. An inhibition of ADAM10 is expected to abrogate cancer growth and metastasis by interfering with the substrate proteolysis and function [61, 62]. We find that fendiline-mediated inhibition of ADAM10 at the membrane protrusions compromises its enzymatic function at the cell surface, rendering it unable to cleave cadherins, which in turn promotes cell adhesion and tight adherens junction formation. This enhances the cadherin-catenin association and stabilization of the adherens junctions, with a subsequent reduction in the levels of β-catenin and TCF/LEF-dependent cyclin D1, c-Myc and CD44 expression. The finding that ADAM10 downregulation significantly reduces the expression of these β-catenin target genes demonstrates the effectiveness of inhibiting ADAM10 activity to combat cancer. Furthermore, it is known that expression of ADAM10 is also regulated by β-catenin/TCF-dependent signaling [63], thereby implying a positive feed-back regulation of tumor growth by this protease and emphasizing the importance of inhibiting ADAM10 in inhibition of cancer progression..

Analysis of human PDAC samples show that expression of both ADAM10 and c-Myc are elevated in PDACs. Our studies show that treatment with fendiline and knockdown of ADAM10 inhibit cyclin D1, CD44 and c-Myc expression, raising the possibility that expression and activation of these three proteins in PDACs may be a reflection of aberrant calcium signaling and ADAM10 activation. Studies in breast cancer cells have shown that expression and activity of c-Myc is regulated by ORAI3 [64], which is upregulated in PDACs (TCGA data, Figure 1A). Further, treatment of cancer cells with SKF96365 showed reduced viability of cancer cells (Figure 1B and 1C) supporting a role for store operated calcium entry in cancer cell survival and proliferation. These results suggest that targeting the altered calcium signaling impairs tumor growth by interfering with expression or function of tumor promoting proteins, such as c-Myc, ADAM10 and β-catenin. Whether specific calcium channels on cancer cells are modulated by fendiline or whether it brings about the effects through interfering with a calcium-dependent or independent pathway is unclear. Since fendiline is known to have antagonistic effects on calmodulin, it is quite possible that a calmodulin-dependent mechanism is abrogated upon treatment of cancer cells with fendiline.

Furthermore, fendiline has been shown to interfere with K-Ras cellular distribution and function in various cancer cells [17]. Our studies show that, in addition to the β-catenin-TCF/LEF-dependent target gene regulation, fendiline also interferes with the vesicular trafficking, recycling and membrane targeting of ADAM10 and its substrates. Calcium influx has been shown to play a role in Rab-dependent vesicle recycling, membrane docking and exocytosis of proteins [65]. Here we demonstrate that Panc1 cells treated with fendiline show APP accumulation in the EEA1 and Rab7 positive early and late endosomes, respectively, but not in the Rab11 positive recycling and exocytosis-associated endosomes, indicative of defective recycling and membrane targeting of APP. Similar results were seen with another substrate of ADAM10, namely Notch (data not presented here). The inhibition of membrane targeting interferes with the cellular functions of APP and Notch, which includes signaling associated with enhanced growth, EMT, migration, invasion and stemness of cancer cells [19, 34, 66-68]. Studies have shown that ADAM10 recycling and membrane targeting is regulated by the small GTPase Rab14 [69]. Therefore, it is possible that the defective membrane association of ADAM10 in fendiline treated cells is brought about by an inhibition of the Rab14-dependent recycling machinery. This suggests that one of the mechanisms by which aberrant calcium signaling enhances cancer cell growth and metastasis could be through enhanced Rab-mediated recruitment of ADAM10 and its substrates to the plasma membrane, where ADAM10 actively cleaves its substrates, such as cadherins, CD44, Notch and APP, to promote growth, EMT, migration, invasion and metastasis of cancer cells. Fendiline treatment led to defective recycling of endocytosed vesicles and accumulation of membrane-targeted proteins in the early endosomes. Thus, it appears that, in addition to interfering with β-catenin-TCF/LEF signaling and target gene expression, fendiline exerts its effects by interfering with small GTPases involved in protein trafficking and recycling, leading to enhanced accumulation of proteins in the trans-Golgi network, thereby causing cytotoxicity and cell death in cancer cells.

All together, the data presented here show that calcium dysregulation induces cancer cell proliferation, migration and invasion through activation of ADAM10, which is a novel and innovative finding in pancreatic cancer (Figure 8L, schematic). Treatment with fendiline prevents ADAM10 activation, and cadherin-catenin signaling and TCF/LEF-dependent gene expression as well as protein sorting and recycling. Although the various calcium channel blockers showed differential efficacy, establishment of fendiline as an effective agent, might open new avenues to combat PDACs, either alone, or in combination with other therapeutic agents.

## MATERIALS AND METHODS

### Cell lines, reagents and transfections

The cells lines (MiaPaCa2 and Panc1) and the culturing conditions were described previously [19]. Fendiline.HCl and Nifedipine were purchased from Thermo Fisher Scientific (Waltham, MA), Diltiazem.HCl, Isradipine, SKF96365.HCl and Gemcitabine.HCl (Gemzar) were purchased from Tocris (Minneapolis, MN). Control non-targeted siRNAs and ADAM10-specific siRNAs were purchased from Santa Cruz Biotechnology (Dallas, TX) and transfected into MiaPaCa2 cells as per our published protocol [19].

### Western blotting and antibodies

The antibody details (company, catalog number, species, and dilution) are provided in Table 1. Cells were treated with the drugs at the indicated concentrations, washed with PBS, followed by lysis in 1X RIPA buffer (150mM NaCl, 1% NP-40, 0.5% sodium deoxycholate, 0.1% SDS and 100mM Tris, pH 8.0) containing protease inhibitor cocktail (Roche, Indianapolis, IN), 1mM sodium fluoride, 1mM sodium orthovanadate and 1mM PMSF. In the case of PDAC tissue samples homogenization was carried out using an electric homogenizer in RIPA lysis buffer. Protein estimation was performed against a BSA standard using the Pierce 660 reagent (Thermo Fisher Scientific, Waltham, MA). 20μg protein was loaded on a 12% SDS-PAGE gel and western blot analysis was carried out as described previously [19].

**Table 1:** Table shows a list of various antibodies used in the studies along with the company name, catalog #, species and dilutions used for western blot analysis

Antibodies	Company & Catalog #	Species	Dilution
Actin	Sigma, St. Louis, MO; A5316	Mouse	1:10 000
ADAM10	Abcam, Cambridge, MA; ab1997	Rabbit	1:1 500
6E10 for Amyloid Precursor Protein (APP)	Covance, Dedham, MA; 39320-200	Mouse	1:1 000
C-APP	Millipore, Billerica, MA; AB5352	Rabbit	1:2 000
c-Myc	Millipore, Billerica, MA; 05-724	Mouse	1:1 000
N-Cadherin	Millipore, Billerica, MA;	Mouse	1:1 000
Cd44	Cell Signaling, Danvers, MA; 3578	Rabbit	1:1 000
Cleaved PARP	Cell Signaling, Danvers, MA; 9541	Rabbit	1:1 000
Vimentin	BD Pharmingen, San Jose, CA; 550513	Mouse	1:15 000
Cyclin D1	Santa Cruz, Dallas, TX; Sc718	Rabbit	1:1 000
Alexa Fluor 488	Invitrogen, Carlsbad, CA; A11105	Mouse	1:2 000
Alexa Fluor 594	Invitrogen, Carlsbad, CA A11029	Rabbit	1:2 000
Goat anti-mouse IgG-HRP	Southern Biotech, Birmingham, AL; 1030-05	Goat	1: 5 000
Goat anti-rabbit IgG-HRP	Southern Biotech, Birmingham, AL; 4010-05	Goat	1:5 000

### Cell toxicity assay

Thiazoyl Blue Tetrazolium Bromide (MTT) (Sigma, St. Louis, MO) was used for the cytotoxicity assay, following our established protocols [19]. Briefly, 3000 Panc1 or MiaPaCa2 cells in 100μl complete medium were plated per well in a 96 well plate for 24h and treated with various calcium channel blockers at concentrations ranging from 1M to 100μM for an additional 24h. Where indicated, gemcitabine was used at a concentration of 100ng/ml. At the end of the incubation MTT was added to the wells at a final concentration of 1mg/ml and incubated for 2h and the purple formazan crystals formed were solubilized in 100μl of isopropanol containing 4mM HCl and 0.1% NP-40 and OD was measured at 540nm.

### Immunocytochemistry

Panc1 cells were plated in 8-chamber slides and treated with fendiline for 24h and staining was carried out following the established protocols [70]. Secondary antibodies used included anti-rabbit Alexa Fluor 594 or anti-mouse Alexa Fluor 488. For staining of actin we used Alexa Fluor 488-labeled phalloidin (Invitrogen, Carlsbad, CA). Slides were mounted using Fuoro-Gel (Thermo Fisher Scientific). Zeiss Axioimager (Carl Zeiss Microimaging GmbH, Gottingen, Germany) was used to observe the slides and analysis was done using Axiovision Rel 4.8 software.

### Analysis of endosomal recycling using FM lipophilic dye

FM 1-43FX was purchased from Invitrogen (Carlsbad, CA) and staining was performed following the manufacturer's protocol. Briefly, cells plated onto poly-lysine coated 8 chamber slides were treated with 5μg/ml FM 1-43FX dye diluted with medium for 5min., and washed and replenished with culture medium with or without fendiline for 6h. At the end of the incubation the cells were washed, fixed with 4% para-formaldehyde for 10min., washed with PBS and mounted using Fluoro-Gel prior to analysis with a Zeiss Axioimager using AxioVision Rel 4.8 software.

### Colony formation assay

To determine the effect of drugs on anchorage independent growth of cells, soft agar assays were performed as described previously [19]. Briefly, a 0.6% sterile agar in growth medium was added to 12-well plate and allowed to solidify at room temperature for 30min., which formed the base layer. For the top layer, growth medium containing 5 000 cells along with the indicated drug concentrations were mixed with the 3% agar solution to obtain a final concentration of 0.3% agar and layered over the base layer in each well. The wells were re-fed with drug containing media once a week to prevent drying and at the end of 2.5 weeks, the colonies were visualized by staining with MTT at a final concentration of 1mg/ml for 4h at 37°C.

### Cell proliferation assay using BrdU labeling

Cells were plated in 8-chamber slides and treated with the indicated concentrations of drugs for 24h followed by labeling with BrdU for 1h at 37°C and analyzed using the BrdU labeling kit from Roche (Indianapolis, IN), following the manufacturer's protocol. BrdU stained slides were mounted using Fluro-Gel and analyzed using Zeiss AxioImager for brightfield microscopy. Proliferation in response to treatment was measured as percent BrdU positive cells from the total number of observed cells in the chamber.

### Cell cycle analysis by flow cytometry

Propidium Iodide (PI) staining followed by Flow cytometry was used to analyze the effect of drugs on cell cycle progression, using our previously published protocol [55]. Briefly, cells were trypsinized and grown for 24hr and treated with the indicated doses of fendiline for an additional 24h, trypsinized and washed twice with PBS and fixed with 70% ethanol. Cells were stored at −20°C for at least 24h, washed twice with PBS and resuspended in 1ml of 1X PBS containing 0.1% triton X-100, 40μg/ml RNase and 20μg/ml propidium iodide followed by 30min incubation at room temperature. PI stained cells were analyzed on BD FACSCanto II flow cytometer (BD Biosciences, San Jose, CA) and cell cycle analysis was performed using the Modfit LT program.

### Migration assay

Migration assay was performed following the established protocol [71, 72]. Briefly, Panc1 cells were allowed to grow to confluence in a 12 well dish, serum starved for 24h and scratch wound was made using a 200μl yellow tip. Cells were replenished with medium containing DMSO (vehicle control) or fendiline 15μM and images of the wound area were taken at 30min., 12h and 24h, at a marked region. Percent migration was calculated based on the cell free area measured at 12h and 24h in relation to that measured at 30min.

### Boyden chamber assay

Panc1 cells were pre-treated with or without fendiline at the indicated concentrations for 2h and 10,000 cells were plated on to the Boyden chamber insert (Thermo Fisher Scientific) placed in the chamber containing DMEM with 20% serum in the presence or absence of the drug. Cells were allowed to migrate for 4-6 hours and fixed with paraformaldehyde. Cells from the top of the chamber inserts were removed using a Q-tip and the migrated cells were stained using hematoxylin or Hoechst, and analyzed using a Zeiss AxioImager.

### Immunohistochemical analysis of human PDAC tissue tumor microarray

The PDAC tissue array was purchased from amsbio (Cambridge, MA). The slides contained duplicate samples representing normal tissue, islet cell tumor, and Stage I, II, III adenocarcinoma with regional or distant metastasis. Immunohistochemistry was performed following our established protocols [73]. Briefly, slides were heated at 60°C and sequentially passed through Xylene, ethanol and dH_2_O to remove paraffin and to rehydrate the tissue sections, respectively. The slides were then heated in 10mM citrate buffer, pH 6.0 at 95°C for 10min. for antigen retrieval, and incubated for 20min. in methanol containing 0.5% H_2_O_2_ to quench non-specific peroxidase activity. Slides were incubated with 10% normal goat serum in TBS containing 0.2% Triton X-100 to block non-specific antibody binding and incubated overnight at 4°C with 1:1000 dilution of ADAM10 antibody. Slides were then incubated with biotinylated anti-rabbit antibody for 1h followed by ABC reagent, as described by the manufacturer's protocol (Vector laboratories, Burlingame, CA) and were developed using the DAB kit from Vector lab. Slides were analyzed and quantified by Dr. Coppola, at Moffitt Cancer Center. The stain was semiquantitatively scored based on the intensity as negative (0), weak (1), moderate (2) and strong (3). In all cases at least 34% of the tumor was positive for ADAM10.

### Statistical analysis

The data generated from cell lines was repeated at least 3 times and quantified using image J image analysis tool. MTT assay and colony formation assay were performed in quadruplicate or triplicate, each time, statistical analysis was performed using *Student's t test.* For the human samples, data from western blot analysis were quantified using Image J image analysis tool and statistical analysis was performed using ANOVA.
